# Antioxidant influence on poultry liver morphology and hepatocyte ultrastructure

**DOI:** 10.14202/vetworld.2019.1716-1728

**Published:** 2019-11-07

**Authors:** Evgeny Skovorodin, Guzel Bronnikova, George Bazekin, Oleg Dyudbin, Roman Khokhlov

**Affiliations:** 1 Department of Morphology, Pathology, Pharmacy, and Non-communicable Diseases, Federal State Budget Educational Institution of Higher Education Bashkir State Agrarian University, Ufa, Russia; 2 Department of Veterinary Medicine, Federal State Budget Educational Institution of Higher Education “Penza State Agrarian University,” Penza, Russia

**Keywords:** antioxidants, diisopropylammonium dichloroacetate, Dironax, ducks, geese, hepatocytes, liver, morphology, quails, selenium, Solvimin Selen, ultrastructure

## Abstract

**Background and Aim:**

The poultry farming development is held back by necessity to use the concentrates with the increased number of crude protein, mycotoxicoses, and subclinical infections concentration. They make a significant impact on the liver, therefore affecting its morphofunctional condition. Antioxidants use can prevent the negative influence of these factors. This study aimed to examine the impact of feed supplements containing natural antioxidants and synthetic antioxidants.

**Materials and Methods:**

The Muscovy ducks, Hungarian White geese, and quails were the study object. Birds after hatching from eggs were split into two groups: Control and two experimental. The control group (40 birds of each species) received a normal diet in accordance with the type and age. The young ducklings, goslings, and quails of the first experimental group (30 birds of each species) received water with diisopropylammonium dichloroacetate (Dironax). The young ducklings, goslings, and quails of the second experimental group (30 birds of each species) received liquid multivitamin preparation, containing organic selenium form (Solvimin Selen) from the 1^st^ day of the postembryonic development to the age of 60 days. We performed the weighing of the young ducklings, goslings, and quails, determined the live weight, liver weight, using the electronic scales (measurement inaccuracy is 0.02 g). To conduct the morphometric, histological, and electron microscopic studies liver, we killed the birds at the age of 1 day, 15 days, a month and 2 months during the postembryonic ontogenesis.

**Results:**

The performed overall studies allowed to determine the positive influence of the antioxidants on growth and development of the meat bird, whose body mass increased by 5-10% in comparison with the control parameters. The antioxidants use prevents the development of fatty, hydropic and parenchymal degeneration, hepatocyte and epithelial cells necrosis of the bile ducts, and connective tissue proliferation with its further fibrosis.

**Conclusion:**

This study proved that it is more effective to use well-digestible, fast-acting natural polyvitaminic antioxidant complex with selenium, starting from the 1^st^ day of the postembryonic ontogeny.

## Introduction

Poultry farming contributes a lot to Russian economics as well as ensures its food safety, providing the domestic market with high-quality animal protein, required for human body health support. With this regard, meat ducks, geese, and quail breeding seem to perspective [1].

Genetics and selection allowed us to increase productivity significantly and to improve feed use. However, new issues put many tasks before specialists in the sphere of poultry farming, feeding, and avian diseases. The key element in maintaining the productive birds’ health is to provide the organ with natural bioregulators – biologically active substances. The in-depth study of the biochemical processes in birds’ organisms and their morphological manifestations contribute to the creation of new types of feed and feed supplements, which best suit each individual species and breed [2-10].

To study the effect of natural antioxidants (selenium and Vitamin E) is a perspective direction in poultry industry [1,4,11-14]. Selenium is an essential microelement, one of the most important indirect action feed antioxidants. It is particularly important for the Republic of Bashkortostan, as we are located in the endemic region, characterized by selenium, iodine, zinc, cobalt, and manganese deficit [15].

Synthetic antioxidants have great possibilities due to their low cost and feeding convenience [16-19]. Diisopropylammonium dichloroacetate is similar to its chemical nature and pharmacological activity to pangamic acid, having a lipotropic effect. It improves the antitoxic liver function and optimizes the animal weight growth, increases the organism resistance, and prevents parenchymal organs alteration [20]. Dipromony, used in medicine (Pat. 369572, Switzerland; Pat. 11405877, Germany; Pat. 862248, Great Britain; Pat. 1295338, France), is quite an expensive medication that prevents from its use for breeding.

Diisopropylammonium dichloroacetate synthesis method (Pat. 2480212, Russian Federation) has been developed in the Republic of Bashkortostan, which allows to obtain cheap diisopropylammonium dichloroacetate of medical quality – Dironax. This makes it possible to use it for animal treatment as a hepatoprotector and as a feed supplement [21]. The impact of this preparation on meat bird has not been studied sufficiently [22].

The morphological study of the liver, using modern techniques (morphometry, histochemistry, and electron microscopy), allows to evaluate objectively the main parameters of metabolism, to reveal organ pathology, which develops as a result of the negative effect of crude protein high level in the diet, mycotoxin intake, and subclinical infections [3,6,19,23-29]. Based on this, we chose the liver as the object for our study as it is the indicator of metabolism level and pathological processes for meat bird during fattening, and antioxidants influence on this organ.

The scientific novelty of this work lies in the fact that for the first time, using a complex of the morphometric, histochemical, and electron microscopic methods, the study was conducted on the liver of Muscovy ducks, Hungarian White geese, and quails during fattening and against the antioxidants use. There has been established the regularities of meat bird growth and development, liver weight growth of these birds against the feed supplements use. Macroscopic and histological changes of meat bird have been described for the first time, and the bird hepatocytes ultrastructure has been studied in detail against the use of the antioxidants. The presented long-term study allows to clarify these issues, to develop specific practical measures for hepatopathy prevention, and to increase meet birds fattening efficiency.

This study aimed to examine the impact of the feed supplements, containing natural antioxidants (Solvimin Selen) and synthetic antioxidant diisopropylammonium dichloroacetate (Dironax).

## Materials and Methods

### Ethical approval

All the experiments were conducted in accordance with the legislation (European Convention for the Protection of Vertebrate Animals; GOST P 53434-2009 – “Good laboratory practice principles;” International Guiding Principles for Biomedical Research Involving Animals (1985); European Convention for the Protection of Vertebrate Animals used for Experimental and other Scientific Purposes; Guide for the Care and Use of Laboratory Animals; Decree of the Ministry of Health of the USSR No. 755 dated August 12, 1977 “About measures on further improvement of the work organizational forms, involving the experimental animals”) and based on the Ethics Committee report of Federal State Budget Educational Institution of Higher Education “Bashkir State Agrarian University” in animals study (No. 8 dated February 22, 2009).

### Animal feeding and management

The Muscovy ducks, Hungarian White geese, and quails, kept in the university’s vivarium, were the study object. Birds are fed balanced combined fodder PK-2 (Table-1), gave the shells. The diet corresponded to the feeding of birds on farms was characterized by high protein content. Drinking was done with the help of drinkers. The temperature of the water in the drinkers was at least 17°C. Feeding, watering, and maintenance of the birds were consistent with the recommendations of the Russian Institute of Poultry farming (“Veterinary rules of the maintenance of a bird in personal farmsteads of citizens and the enterprises of open type” (are approved by the order of the Ministry of Agriculture of Russia of April 3, 2006 No. 103), “Recommendations for feeding poultry” – Russian Institute of Poultry farming, Sergiev Posad, 2016).

**Table-1:** Dietary ingredients and nutrient content of experimental diets.

Ingredients	
Wheat, %	43.72
Corn, %	30.41
Soybean meal, %	11.15
Sunflower meal, %	8.0
Fish meal, %	1.80
Lysine, %	0.45
Feed methionine, %	0.2
Sodium chloride, %	0.24
Monocalcium phosphate, %	1.33
Limestone, %	1.61
Premix (P_5-1_), %	1.00
Chemical compositions	
Feed unit	116.20
Exchange energy, MJ/kg	287.56
Crude protein, %	18.00
Crude fiber, %	3.50
Lysine, %	1.10
Methionine+cystine, %	0.86
Sodium chloride, %	0.30
Phosphorus, %	0.70
Calcium, %	0.95
Crude fat, %	3.05

### Animals, experimental design, and treatments

Birds after hatching from eggs were divided into two groups: Control and two experimental. The control group (40 birds of each species) received a normal diet in accordance with the type and age. The young ducklings, goslings, and quails of the first experimental group (30 birds of each species) received water with diisopropylammonium dichloroacetate synthesized in Bazis LLC (Ufa) – Dironax. The young ducklings, goslings, and quails of the second experimental group (30 birds of each species) received liquid multivitamin preparation, containing organic selenium form – Solvimin Selen from the 1^st^ day of the postembryonic development to the age of 60 days.

### Morphological methods

We used the contemporary objective morphological methods of study: Anatomical (body weight dynamics, weighing, preparation, and anatomical features description of the Muscovy ducks, Hungarian White geese, and quails organs), organometric (organs weight and size), histological, histochemical, and electron microscopic methods of morphological analysis. We performed the weighing of the young ducklings, goslings, and quails, determined the live weight, liver weight, using the electronic scales (measurement inaccuracy is 0.02 g). To conduct the morphometric, histological, and electron microscopic studies, we killed the birds at the age of 1 day, 15 days, a month and 2 months during the postembryonic ontogenesis. Experimental birds killing periods were determined based on the critical phases of the bodyweight growth and liver formation [30,31].

To study the microscopic changes in the liver of the experimental and control birds, the liver parts were fixed in 10% neutralized formalin, Buen and Carnoy liquids, embedded in paraffin blocks. The paraffin sections (5 µm) were colored with hematoxylin and eosin to detect glycogen and neutral glycosaminoglycans according to McManus [32]. The frozen liver sections were colored with Sudan III to detect the neutral lipids and Sudan Black B to detect phospholipids. To conduct the electron microscopic study, the parts were fixed in cooled 2.5% glutaraldehyde based on cacodylate buffer solution (pH 7.2-7.4) and postfixed in 2% O_s_O_4_ solution based on the same buffer, dehydrated in ethanol with increasing concentration and poured into Epon-812. The ultrathin sections were prepared on LKB-III 8800 Ultratome (Sweden), contrasted with 2% uranyl acetate aqueous solution, and lead citrate [33] and examined in JEM-1011CX II transmission electron microscope (JEOL, Japan).

### Morphometry and statistical analysis

We used Brodie formula to determine the relative increase in body weight and liver. 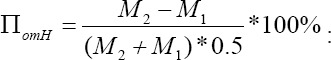 Where W_1_ – weight at the beginning of the period, W_2_ – weight at the end of the period. This indicator allowed to determine the weight’s “relative growth speed” and to compare the equal values in different bird’s species. ImageJ program was used for the cytometric studies [34]. The nuclear-cytoplasmic ratio was determined by dividing the nucleus volume by the hepatocyte volume. Student’s t-criterion was used to determine the statistical validity of the indicator. Due to the relatively small number of groups and deviations from normal distributions of variables, a Kruskal-Wallis non-parametric ANOVA test was performed with licensed software package STATISTICA 10. The level of the significance was fixed at p<0.05.

## Results

### Body and liver weight

The relative birds’ weight gain is moderate in the first 15 days and reaches 90-110%. It reaches its maximum in the following 15 days and amounts to 150-180%. This indicator reduces to 60-80% by the end of the experiment. We found out that the antioxidants use aids to increase the meat bird body weight gain in comparison with the control groups by 5-10%. Such an effect is highly important for the use of the feed supplement, containing selenium (the difference is significant at p<5%).

The liver weight increases synchronously in relation to body weight growth. The relative liver weight gain is moderate within the first 15 days after hatching. The period from 15 to 30 days is characterized by the maximum relative organ’s weight gain, especially with regard to the quails. The relative liver weight gain reduces twice (geese and ducks) and thrice (quails) by the 60^th^ day. During these three stages, Solvimin Selen has the greatest effect on the liver growth rates compared to Dironax, but the difference between the experimental groups is not significant (at p<5%).

### Microscopic changes in the liver on 1^st^ day

The control group birds had the following morphological changes. During the external examination, the liver of the 1-day-old ducklings, goslings, and quails has an ocher-yellow color. The organ’s lobed structure is not expressed. The stroma is represented by poorly distinguishable thin collagen fibers. The relative liver parenchyma volume is maximum in relation to the stroma of the hatched chicks, compared with the subsequent age periods. It creates the impression of fatty organ dystrophy.

During the microscopic examination, especially when fixing the liver in Karuna fluid, hepatocytes cytoplasm has a foamy appearance, somewhat vacuolated (Table-2). The glycogen content in the cytoplasm is not significant. Therefore, you can make a wrong conclusion that these cells are subject to protein-fatty degeneration. It is likely an artifact. This effect is not pronounced when fixing in Buen liquid or when fixing with gradual formalin concentration increase from 3% to 10%. We did not observe such a situation during prefixing in glutaraldehyde and coloring of the semifine sections. Sudan Black B positive coloring means that these vacuoles are primarily presented by phospholipids.

**Table-2:** Liver morphological changes of the day-old bird (10 birds per group).

Type of morphological changes	Number of the birds with changes		

	Ducks	Geese	Quails
A. Cytoplasm vacuolization	10	10	10
B. Parenchymatous dystrophy	2	0	1
C. Fatty dystrophy (Sudans)	2	1	1
D. Connective tissue proliferation	0	0	0
E. Extracellular dystrophy and stroma fibrotic changes	0	0	0
F. Nuclear-cytoplasmic ratio	0.046±0.002	0.038±0.001	0.072±0.005

Besides, 1-2% of chicks (Table-2) suffered from cytoplasm swelling and clouding as well as from appearance and accumulation of small acidophilic protein granularity in the cytoplasm. Herewith, the cell boundaries and the nuclei outlines are hardly distinguishable. A different hatched chicks’ maturity level and the protein metabolism level explain it.

**Figure-1:**
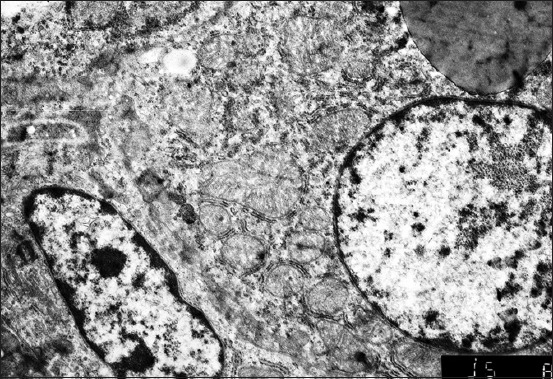
Hepatocyte and endotheliocyte of a 1-day-old duckling. Nucleus, hepatocyte perinuclear zone with a lipidic drop. Macrophage. Enlarged 10,000 times.

It was started with the electron microscope that the hepatocytes nuclei are located eccentrically. The external and internal shell membranes are separated by a wide space. It is uneven, with extensions and narrowings around the nuclear pores, foamed. There are many ribosomes on the nucleus shell from the side of cytoplasm. Nuclei chromatin is marginal, in the center – one, rarely two nucleoli. Nucleoplasm contains fine chromatin (Figure-1). The hepatocytes nuclei of the 1-day-old birds contain nucleoli with prevailing granular component and small areas of dense fibrillary component.

The granular cytoplasmic reticulum (GCR) of the 1-day-old birds’ hepatocytes is represented by flat cisterns, in the form of tightly packed membranes. They are located around one of the mitochondria sides as a rainbow. The round-shaped hepatocyte mitochondria of the 1-day-old birds are located mainly near the nucleus and in the sinusoidal or biliary field. Mitochondria have a small number of cristae; it means that the anaerobic respiration prevails (Figure-1). Mitochondria of some hepatocytes are enlarged, their membranes are stretched, stratified, and the scallops are unevenly thickened and shortened, the matrix becomes clear with the appearance of transparent vacuoles (mitochondria vacuolization).

### Microscopic changes in the liver on 15^th^ day

The relative liver parenchyma volume of all the studied bird species out of the control group decreases slightly due to the connective tissue proliferation of the organ’s stroma by the 15^th^ day after hatching. We observed small gaps of blood vessels, with their increased blood fullness. It is caused by the histion blood circulation optimization. The hepatic lobule architectonics become pronounced due to the dark hepatocytes along the periphery and connective tissue proliferation (Table-3).

**Table-3:** Liver morphological changes of 15 days birds (10 birds per group).

Type of morphological changes	Group	Number of birds with liver changes		
	
	Birds species	Ducks	Geese	Quails
A. Cytoplasm vacuolization	Control	8	6	8
	Dironax	6	5	4
	Solvimin Selen	4	3	4
B. Parenchymatous dystrophy	Control	4	4	3
	Dironax	2	2	2
	Solvimin Selen	1	1	1
C. Fatty dystrophy	Control	3	4	2
	Dironax	2	2	1
	Solvimin Selen	1	1	1
D. Connective tissue proliferation	Control	6	8	7
	Dironax	4	3	4
	Solvimin Selen	3	4	2
E. Extracellular dystrophy and stroma fibrotic changes	Control	5	4	6
	Dironax	2	1	1
	Solvimin Selen	1	2	2
F. Nuclear-cytoplasmic ratio	Control	0.049±0.003	0.041±0.002	0.078±0.006
	Dironax	0.051±0.004	0.043±0.003	0.076±0.006
	Solvimin Selen	0.054±0.004*	0.046±0.003*	0.079±0.007*

* p<0.05

Hepatocytes contain large bright nuclei with clear marginal chromatin in the form of a thin strip along the edge. About 30-40% of the control group birds had intracellular dystrophy signs, which were more pronounced than for the 1-day-old birds. About 2-4% of the birds have small-sized obesity of the cytoplasm of the hepatocyte. The stroma’s connective tissue and vessel walls of some birds (4-6 %) became basophilic due to metachromasy. In this area, lymphoid nodules appeared.

**Figure-2:**
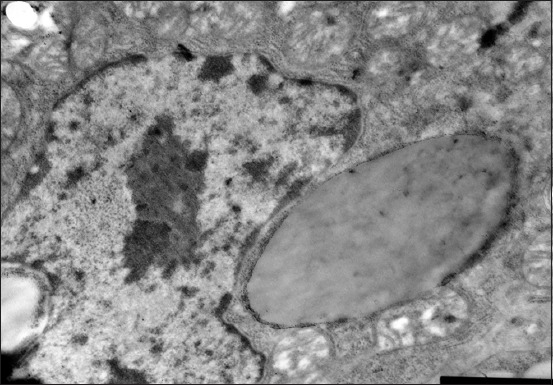
Hepatocyte of a 15-day-old gosling of the control group. The lipidic drop in the cytoplasm perinuclear zone. Enlarged 31,000 times.

The nucleoli are larger in comparison with the 1-day-old birds (Figure-2). The hepatocyte mitochondria content increases. The granular and smooth cytoplasmic reticulum ratio increases at this age. The glycogen granules number increases and they are located in separate cytoplasm parts. Lipids get a typical structure of the rounded droplets with banded electron density (Figure-2); it means that the triglycerides prevail in them, as proved by the stain of Sudan III (Table-3).

**Figure-3:**
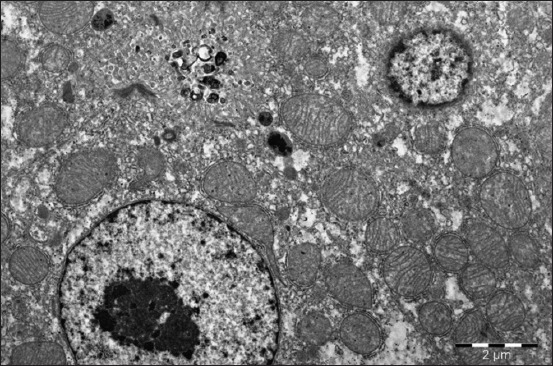
Hepatocyte of 15-day-old goose of the experimental group (selen). Hepatocyte biliary field with a large light nucleus and reticular nucleole, plenty of mitochondria with stretched cristae. Enlarged 10,000 times.

The liver structure of the birds received Dironax is characterized by an increase in the relative stroma volume due to the vascular component severity and connective tissue proliferation by the age of 15 days (Table-2), although the latter is less pronounced compared to the control (Table-2). Hepatocytes become polygonal. Their cytoplasm is homogeneous, turbid. Some cells located on the periphery of the lobules contain small drops of neutral fats (Table-2). The nuclei are located mainly in the center (Figure-3). Lymphoid cells gather around the vessels and triads.

**Figure-4:**
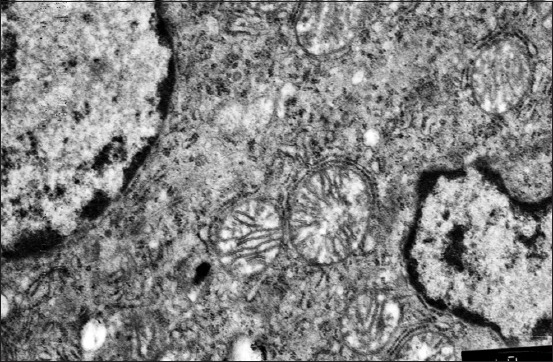
Hepatocyte nucleus and perinuclear zone of a 15-day-old duckling of the experimental (selen). The optimal structure of a nucleus, mitochondria surrounded with granular endoplasmic reticulum, plenty of free ribosomes. Enlarged 21,000 times.

The liver parenchyma volume of the birds received Solvimin Selen decreases insignificantly by the age of 15 days. Neutral hepatocytes fat drops are expressly decreased in number and size. The nuclei are located mainly in the center (Figure-4).

### Microscopic changes in the liver on 30^th^ day

The relative parenchyma volume of the control group birds significantly decreases by the age of 1 month due to increase in the hepatocytes sizes, and not in their number, stroma proliferation, changes in sinusoids, and extracellular spaces size. The organ gets more central veins and triads. Apparently, this is due to the formation of new hepatic lobules, organ’s connective tissue proliferation and fibrotic changes of the stroma, vessel walls and bile ducts (Table-4).

**Table-4:** Liver morphological changes of 30 days birds (10 birds per group).

Type of morphological changes	Group	Number of birds with liver changes		
	
	Birds species	Ducks	Geese	Quails
A. Cytoplasm vacuolization	Control	9	8	9
	Dironax	4	3	3
	Solvimin Selen	2	2	2
B. Parenchymatous dystrophy	Control	8	9	7
	Dironax	1	2	1
	Solvimin Selen	0	1	0
C. Fatty dystrophy	Control	7	8	6
	Dironax	1	1	1
	Solvimin Selen	0	0	0
D. Connective tissue proliferation	Control	8	9	8
	Dironax	2	3	2
	Solvimin Selen	3	4	2
E. Extracellular dystrophy and stroma fibrotic changes	Control	7	6	8
	Dironax	1	1	0
	Solvimin Selen	0	1	0
F. Nuclear-cytoplasmic ratio	Control	0.044±0.003	0.031±0.002	0.059±0.004
	Dironax	0.048±0.004	0.039±0.003*	0.071±0.006*
	Solvimin Selen	0.049±0.005*	0.038±0.003*	0.073±0.006*

* p<0.05

Almost all the birds suffered from cytoplasm vacuolization, hepatocyte protein-fatty degeneration, and coagulation of the main substance in the triad area, swelling, and partial disintegration of collagen fibers, and plasmorrhagia with impregnation of connective tissue with an eosinophilic substance (Table-4).

**Figure-5:**
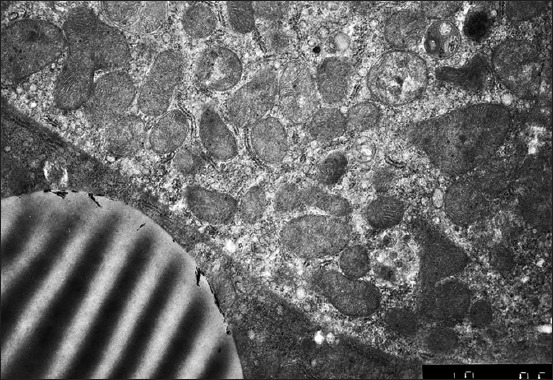
Hepatocyte of a 1-month-old duckling. Prominent signs of hepatocyte protein-fatty dystrophy. Enlarged 31,000 times.

Hepatocytes nuclei have different sizes and round shape. One or two large nucleoli with a reticular structure are located in the nuclei (Figure-5). The hepatocytes cytoplasm with small vacuoles is granular, contains neutral lipids. There are many hepatocytes containing medium and large size lipid droplets on the periphery of the lobules. Hepatocyte contours are not clear. Cells are swollen and enlarged. The degree of hepatocyte swelling is not even. This process is more pronounced far from the central vein. The nature of changes in the cytoplasm and nuclei of epithelial cells is established with a significant increase (Figure-5).

The goslings at this age have the hepatocytes, which are quite enlarged, with signs of granular and fatty dystrophy. This is manifested by a weak cytoplasm granularity and availability of small vacuoles, which, when stained with Sudan, are colored as neutral fats. Hepatocytes cytoplasm vacuolization mainly occurs under the organ’s capsule. Hepatocytes of 30-days goslings have mitochondria with a diameter of up to 1 µ and a round shape. More often they are located closer to the nucleus, and only sometimes closer to the hepatocyte sinusoidal or biliary pole. Hepatocyte mitochondria are typical, with a small number of narrow, parallel small cristae, which comes from the inner membrane and extend toward the matrix. They do not completely block the mitochondria cavity and do not violate the matrix continuity, filling it. Mitochondria matrix is granular, electron-dense.

During the electron microscopic examination, you can clearly see the highly expanded cells of the GCR in the immediate vicinity of the hepatocyte nucleus as for the ducks. The mitochondria are swollen and lack cristae; this process is strongly pronounced in the cell periphery closer to the cytoplasmic membrane. The cytoplasm matrix has a form of a narrow strip between the expanded cells. Here, you can also find the relatively small cells of the GCR. Small lysosomes, having an oval shape in black, are located closer to the cytoplasmic membrane.

There are few glycogen granules. They are located in separate parts of the cytoplasm. Hepatocyte lipids have a form of round average drops. They have a specific bandy electron density due to the predominance of ballast triglycerides and neutral fats.

The hepatocytes of the quails have the form of a 5-10 µm polygonal cell. Sinusoidal and biliary poles differ in the organelles arrangement. A great mitochondria number characterizes the cytoplasm of the hepatocytes. They have a round or an elongated shape with 1-1.5 µm diameter and longitudinal sections length up to 3 µm. You can find dumbbell-shaped, probably dividing mitochondria. However, some hepatocytes have mainly round shape mitochondria and some – elongated up to several microns. It is explained by the mitochondrial network unidirectionality in the elongated hepatocytes and the dense arrangement of mitochondrial cords, which is common for the quail liver.

Mitochondria have a specific structure. Inside, they have a significant number of extended, semicircular, short, eccentrically located cristae, which do not reach the middle of the organelle. Some of the cristae look light against the electronic dense granular matrix, and dark with light center against the light matrix in the mitochondria. Light hepatocytes more often have small size mitochondria with many small cristae. We examined a large number of the mitochondria and the biggest value of these ultrastructures in large cells with a large light nucleus and granular type nucleoli. Binuclear hepatocytes have more mitochondria and they are larger.

The quail hepatocytes have a well-developed cytoplasmic reticulum. It appears in the form of the limited membrane tubules and vesicles. The GCR is covered with ribosomes. The flat cytoplasmic reticulum (FCR) does not have ribosomes. GCR-FCR ratio is different depending on the cell functional state and organism’s specific peculiarities.

The quails GCR is represented mainly by the flat cisterns, most often located close to the mitochondria, circling them partially or semi-circling and forming a kind of a “cap,” or around the mitochondria in the form of a ring. The latter situation is more typical for the binuclear hepatocytes. If you can see the elongated mitochondria in the section, the GCR profiles are located close to them in the form of the cords. The mentioned picture is a specific peculiarity of the quail hepatocytes. The quail hepatocytes GCR in comparison with the mammals, is fragmented, does not form piles of the parallel located membranes.

Besides, the quail hepatocytes cytoplasm includes: Lysosomes, peroxisomes, lamellar complex, filamentous structures, lipids, and glycogen. The nucleus and its structures and nucleolus are the most informative for assessment of the hepatocyte’s functional state.

The liver parenchyma volume of the 1-month birds, received Dironax, significantly decreases, but this value remains higher in comparison with the control. Lipids drop, small turbid granularity of the cytoplasm of the hepatocytes is practically absent or present in 10% of the birds. Against the connective tissue proliferation, 10% of ducks and geese had the signs of stromal-vascular dystrophy (Table-4).

**Figure-6:**
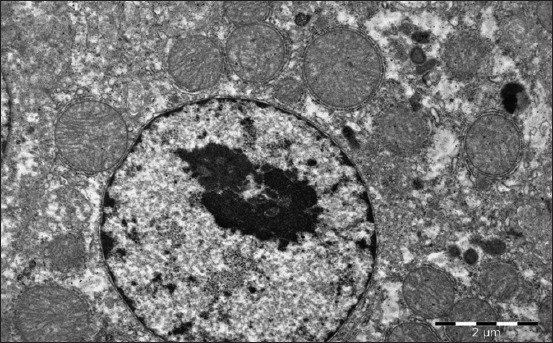
Liver of a 1-month-old goose of the experimental group (Dironax). Hepatocyte with a large light nucleus, plenty of prominent stretched cristae in mitochondria, surrounded with granular endoplasmic reticulum. Enlarged 10,000 times.

You can clearly define finely dispersed chromatin with a low electron density, located diffusely in the nucleus. Chromatin, having a high electron density, is clearly defined in the form of marginal heterochromatin. Electron dense lumps become larger. The GCR is well defined and evenly distributed throughout the cell. Its cisterns are split and even fragmented; they do not form the piles of parallel located membranes. There is a large number of ribosomes on these membranes. The FCR profiles become smaller at the age of 1 month. They are located closer to the cell periphery (Figure-6).

Hepatocytes of the ducklings in this experimental group received selenium have more mitochondria. They have an elongated or incorrect shape with peculiar branches, contain a greater number of radially located cristae; their electron-dense matrix has the form of granules. The number of intramitochondrial cristae increases.

The liver of the 1-month ducklings in this group has a larger number of “dark” hepatocytes, which are responsible for the synthetic activity. In comparison with “light” cells, they contain relatively more developed granular endoplasmic reticulum and few elements of the flat endoplasmic reticulum. They have more free ribosomes and polysomes, their canalicular apparatus is better developed, and the cytoplasm is richer in glycogen. They have a better-developed mitochondria system. They are larger and outnumbers the same amount in “light” hepatocytes by 2-3 times.

**Figure-7:**
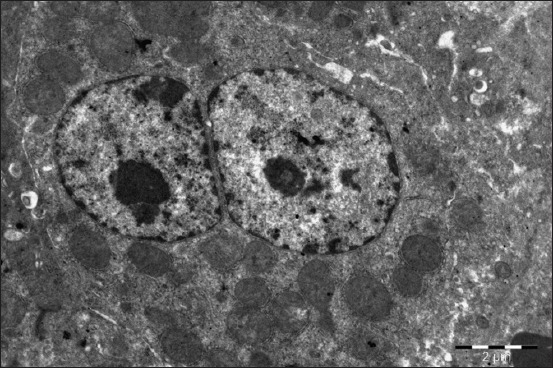
Liver of a 1-month-old duck of the experimental group (selen). Binuclear hypertrophied hepatocyte. Enlarged 10,000 times.

The liver structure of the birds treated with selenium is characterized by a clear architectonics. All the tissue structures are pronounced, well developed, and do not have atrophic, dystrophic changes, signs of the impaired blood and lymph circulation. Hepatocytes contain large light nuclei with a clear chromatin rim around the nucleus periphery and a rounded structured nucleus in the center. Some hepatocytes contain 2-3 small size nuclei. You can often meet the hypertrophied binuclear hepatocytes (Figure-7). Cytoplasm, when stained with hematoxylin and eosin, has a fine-grained structure and does not contain neutral fats. The parenchyma volume reduces insignificantly against connective tissue proliferation.

### Microscopic changes in the liver on 60^th^ day

By the age of 2 months, the birds’ liver of the control group can have a definite structure. The liver parenchyma volume is reduced not only due to the connective tissue proliferation but also due to the fibrotic changes, especially in the triad area around the bile ducts and blood vessels. These figures are lower in comparison with all the preceding age periods (Table-5).

**Table-5:** Liver morphological changes of 60 days birds (10 birds per group).

Type of morphological changes	Group	Number of birds with liver changes		
	
	Birds species	Ducks	Geese	Quails
A. Cytoplasm vacuolization	Control	10	10	9
	Dironax	2	2	1
	Solvimin Selen	1	0	1
B. Parenchymatous dystrophy	Control	9	8	8
	Dironax	0	1	0
	Solvimin Selen	0	0	0
C. Fatty dystrophy	Control	8	9	8
	Dironax	1	2	1
	Solvimin Selen	0	1	0
D. Connective tissue proliferation	Control	10	10	10
	Dironax	2	2	1
	Solvimin Selen	3	4	2
E. Extracellular dystrophy and stroma fibrotic changes	Control	8	7	9
	Dironax	1	1	2
	Solvimin Selen	0	1	0
F. Nuclear-cytoplasmic ratio	Control	0.043±0.003	0.029±0.002	0.053±0.004
	Dironax	0.042±0.003	0.034±0.002*	0.064±0.005*
	Solvimin Selen	0.046±0.005*	0.032±0.002	0.069±0.007*

*p<0.05

Collagen fibers of the 2-month-old birds are thickened. At the same time, there are many thin newly formed reticulated fibers. The stroma has fibrotic changes; the walls of the vessels are thickened with signs of mucoid swelling of the connective tissue. It is caused by the congestions. There are lymphoid cells accumulations in the form of follicles along the blood vessels. The structures of the local immune system are lymph nodules; they have a small size without connective tissue shells.

**Figure-8:**
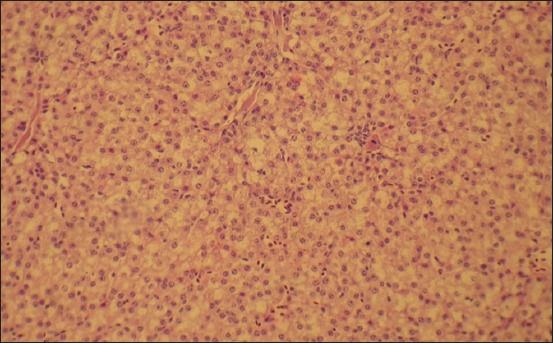
Liver of a 2-month-old quail of the control of hepatocytes (lipophanerosis) in each zone of the organ. Enlarged 400 times.

**Figure-9:**
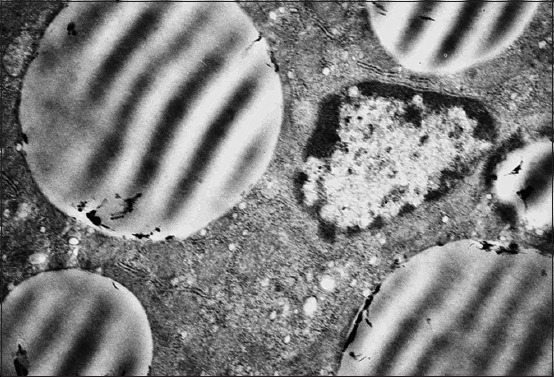
Hepatocyte cytoplasm of a 2-month-old duckling of the control group. Drops of neutral fats around a pycnotic nucleus. Enlarged 31,000 times.

The “glomerular” liver structure is not defined, but there is a pronounced decomposition of the cellular parenchyma elements. Vascular lumens are narrowed because hepatocytes swell. The cytoplasm is reticulated (Figure-8). These are the fat droplets of various sizes, dissolved during histological processing, and stained with Sudan III in frozen sections. Bridges between the cells are the cytoplasm remnants with membranes in a state of lipophanerosis. The liver cells nuclei are located, as a rule, in the center, but they are modified: More often, they are a little reduced in volume, more rarely – wrinkled with incorrect angular shape (Figure-9).

**Figure-10:**
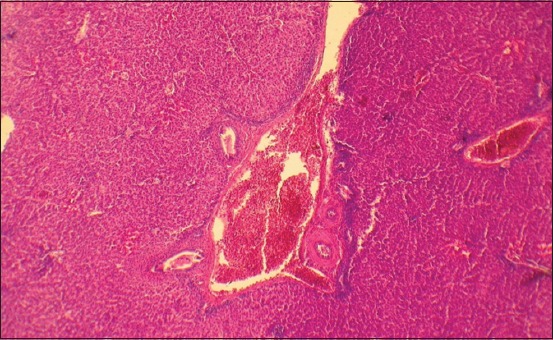
Liver of a 2-month-old goose of the control group. Venous hyperemia. Fibroid changes in liver blood vessels and stroma. Hematoxylin and eosin stain. Enlarged 100 times.

By the age of 2 months, the liver of the control group geese is characterized by a differentiated structure, the collagen fibers reticulum is thick, and the fibers have a close relationship with each other and the intercellular organ components that prove the development of fibrotic changes in the stromal vascular organ component (Figure-10). The hepatic plates’ structure is destroyed; there is a pronounced disconnecting of the parenchyma cellular elements. Vascular lumen is narrowed. Cytoplasm reticulation and granularity are very pronounced around the nucleus.

There observed significant mitochondria changes during the electronic microscopic hepatocytes study. You can often find incoherent conjugated mitochondria in hepatocytes – swollen, incoherent, and edematous mitochondria, which are damaged by membranes. More seldom, you can find weakly conjugated mitochondria. They are large, the cristae pattern is smoothed, and there are extensive membranes destructions and matrix clearing.

The endoplasmic reticulum and ribosomes change. The granular endoplasmic reticulum is hyperplastic, agranular – fragmented with extended profiles or with reduction signs. Some cells get swollen and are subject to myelin figure’s appearance.

The structure of the 2-month-old birds, treated with Dironax, is preserved due to clear glomerular organ organization, characterized by a clear architectonics and a structural component ratio. Lymphoid nodules are not large, structured.

The stroma is well defined. About 10-20% of birds have hepatocyte cytoplasm vacuolization, signs of parenchymal dystrophy, and neutral fats drop. Fibrous changes and stromal vascular dystrophy are not diagnosed. The relative parenchyma volume is significantly higher in comparison with the control. Hepatocyte nuclei are large light with heterochromatin located along the nucleus periphery. The nucleus is small reticular; it is located in the center. The intralobular sinusoidal capillaries are clearly expressed with single red blood cells in the lumen. Hepatocytes have a multi-sided form. The cytoplasm is slightly granular without vacuoles.

**Figure-11:**
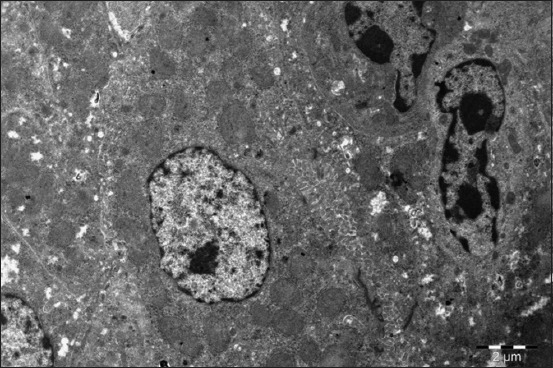
Liver of a 2-month-old duck of the experimental group (Dironax). Hepatocyte with lots of mitochondria and intercellular communication, two macrophages. Enlarged 8000 times.

The hepatocytes nuclei are large, light, with a large centrally located nucleolus, having five components: Granular, fibrillary, dense fibrillary, chromatin, and protein mesh matrix. The nuclei are located a bit eccentrically (Figure-11).

The liver parenchyma volume of 2 months old Muscovy ducks, received selenium-containing supplement, is a little lower than for the control group. The average nuclear plasma ratio of the birds in this group is: For ducklings 0.046±0.005; for goslings 0.032±0.002; and for quails 0.069±0.007. This is significantly lower in comparison with the preceding age periods of the quails, but significantly higher in comparison with the group, treated with Dironax.

**Figure-12:**
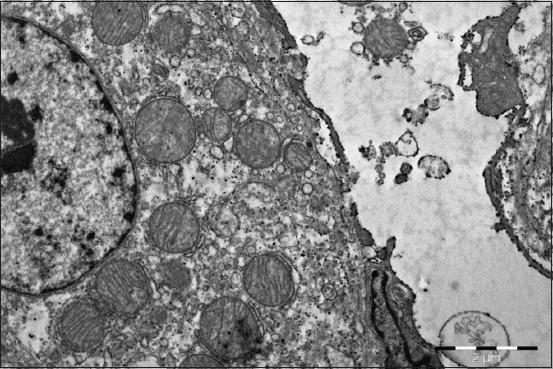
Liver of a 2-month-old duck of the experimental group (Selen). Light hepatocyte with round hypertrophied mitochondria, sinusoid lumen with “active endotheliocytes.” Enlarged 10,000 times.

The hepatocytes have the following ultrastructural differences in comparison with the control group. The cytoplasm has more mitochondria. Mitochondria have short cristae and an enlightened matrix, round shape. Some of them are moderately swollen that indicates the activation of their volatile functions. Cytoplasmic reticulum enlarged cavities form many bright bubbles and cisterns. The GCR canaliculi are located around the mitochondria. There are few glycogen granules compared to the control and they are more dispersed. The hepatocytes nuclei are light, with one or two well-organized nucleoli. The fine chromatin proportion significantly increases, and a part of heterochromatin gets lower (Figure-12). It is likely connected with a higher differentiation degree and synthetic activity. Herewith, it is typical for the geese of this experimental group to have an increased number of glycogen granules in the cytoplasm of the hepatocytes. The number of neutral fats containing drops reduces.

## Discussion

### Microscopic changes in the liver on 1^st^ day

The peculiarity of our study was that we began to study the liver morphology of the 1-day-old meat birds’ chicks. According to the literature, after hatching, there begins a critical phase, associated with a sharp transition from an endogenous nutrition type, which is performed by the egg lipids. Further nutrition is based on the proteins and carbohydrates, received with the feed [31]. In this case, we can observe a significant structural liver change as evidenced by the studies and other authors [3,25,26,35].

According to our data, after performing macro- and microscopic, electron microscopic liver examination of the 1-day-old ducklings, goslings, and quails, there are morphological signs of the incomplete differentiation of the parenchymal elements. At the same time, hepatocytes have high synthetic activity, performed not only due to the elements, received from the outside, but also mainly due to the liver cells’ endogenous nutrient reserves. These very endogenous substances create the impression that hepatocytes are vocalized when stained with hematoxylin and eosin. Hepatocytes of 1 day-old ducklings are exposed to the active metabolic and energy processes, which are performed, first of all, due to phospholipids and lipoproteins, which we identified using the histochemical reactions.

Gesek *et al*. [25] studied the liver of different genetic broiler chickens on the 3^rd^ day after hatching and found that 100% of birds have vacuolar and fatty hepatocytes degeneration, and 80% of the chickens of the genetic line Rossa 308 have venous stagnation. The same data were obtained by Grishina and Baimishev [35], examined the broiler chickens liver, Hamodi *et al*. [26] studied the liver of three birds species. This is compliant with the data of our studies conducted on the meat birds (ducks, geese, and quails).

Therefore, it is necessary to apply a rapidly digestible and fast-acting microelement, having antioxidant action, selenium, starting from the 1^st^ day of existence. Endogenous antioxidants of direct and indirect action type as thioctic (lipoic) acid can also be effective.

### Microscopic changes in the liver on 15^th^ days

Fat, carbohydrate, and energy exchanges undergo significant changes during the further age periods. According to our data, the relative parenchyma volume of the control group reduces several times by the 15^th^ day after hatching. It means that the organism overcame the adaptation period to the environmental factors. Hepatocytes cytoplasm fat content reduces. Lipids are mainly located in the hepatocytes of the peripheral and middles lobules zones. Therefore, fat intense hydrolysis takes place after hatching; it is metabolized as an energetic material due to the increased organ’s functional activity. The number of glycogen granules, located in separate parts of the hepatocyte cytoplasm, increases slightly by the 15^th^ day. Lipids in the hepatocyte have the form of round drops and acquire a typical structure with a banded electron density at this age that means that the triglycerides prevail therein. About 30-40% of the control group birds had the signs of the parenchymal dystrophy. We detected small-drop fattiness in the hepatocytes cytoplasm of 2-4% birds and a mucoid swelling of the connective tissue of the organs stroma and vessels of 4-6% birds.

Our data coincides with the study results of the other authors;

According to Gesek *et al*. [25] by the 10^th^ and 17^th^ days of postembryonic ontogenesis in broilers’ liver, we diagnose the parenchymal degeneration and vacuolar degeneration, venous hyperemia, bile duct proliferation, endothelial cell hypertrophy, and arterial muscle cells hypertrophy. Only half of the chickens had fat degeneration, lymphoid cell infiltration of the surrounding bile, and blood vessels connective tissue. The authors explain this by the subclinical infection caused by *Clostridium perfringens* and viruses, which lead to cholangiohepatitis as well as by aflatoxins, coming with the feed [29,36-38].

### Microscopic changes in the liver on 30-60 days

By the age of 1 month and even more so by 2 months, the control group birds undergo an increase in the liver weight, accompanied by cell hypertrophy prevailing over their proliferation that is consistent with the literature data [29,35]. This is proved by the nuclear-cytoplasmic ratio calculations. The hepatocytes and nuclei volume increased, but the nuclear plasma ratio decreased, which means a decrease in the hepatocytes’ functional activity compared with the previous age. The birds grown in conditions of selenium deficit in soils and water can face the development of granular and fatty liver degeneration by the age of 2 months, which is manifested not only by structural organ changes but also by low growth and development rates of the whole organism.

The relative stroma volume significantly increases. The increase of the stroma proportion in the section is externally manifested at low magnification by well-defined connective tissue structures located in the region of the triads. Stroma fibrous changes in the organ become pronounced. Lymphoid infiltrates and hemorrhages resembling pockets of extramedullary hematopoiesis are found in the liver parenchyma.

According to Jafargolipour *et al*. [19] by the 45^th^ day, the quails get fatty dystrophy, hepatocytes necrosis against high crude protein level of the diet. Osičková *et al*. [27] described birds’ liver histopathology under oxidative stress. According to Paskova *et al*. [17], after examination of the quail liver, stated that this organ, performing the detoxification function, is the first to be affected under oxidative stress. Vitula *et al*. [39] received the same data at quail mycoplasmosis.

Japanese quails are very sensitive to fatty liver hemorrhagic syndrome, and a combination of the lipotropic and antioxidant nutritional supplements has a protective effect against this disease [23]. Holovská *et al*. [40] study showed that in Turkey the use of zinc as the antioxidant had a protective action against the harmful effects of cadmium on the liver cells. Selenium is the factor; protecting liver, heart, and aorta from the obesity risk in rats and quails received a high-fat diet [24,41].

### Morphogenetic mechanisms and the type of hepatosis

In our opinion, dystrophy changes are followed by membrane lipid oxidation. The accumulation of the endogenous metabolites untypical for bird’s lipids is the cellular manifestation of the metabolic disorders because of their insufficient (incomplete) utilization. It should be emphasized that poorly pronounced lipid accumulation does not affect the cell function, and pronounced lipid accumulation can disrupt the cell function and irreversibly damage the intracellular processes by membrane lipid peroxidation.

Based on our electron microscopic analysis, we can make a conclusion that it is lipofanerosis, rather than infiltration and transformation, that plays a predominant role among the morphogenetic mechanisms contributing to the development of the changes typical for dystrophies. Subsequently, the cell membranes’ lipid peroxidation increases according to the “vicious circle” type. The second visible manifestation of metabolic disorders is the lipid accumulation and their insufficient utilization against the gluconeogenesis decrease. Probably this is the reason for the peroxide’s high level to appear in the cells [7], as long as the birds were grown in the endemic zone with selenium deficit [15]. Weak lipids accumulation does not affect the cell’s function. However, the neutral fats pronounced accumulation by the age of 2 months deranges the hepatocyte’s function that irreversibly damages ultrastructures and intracellular redox processes.

According to Gesek *et al*. [25], more often the ultrastructural disorders are typical for mitochondria and granular endoplasmic reticulum. Mitochondria underwent swelling; we detected polymorphism, proliferation, and membrane damage. Almost all mitochondria look like dense bodies. The GCR was subjected to defragmentation or acinar transformation.

### Effect of biologically active additive

It should be noted that the use of complex selenium-containing preparation Solvimin Selen and hepatoprotector Dironax adjusts the established patterns of the liver organogenesis. The selenium-containing preparation optimizes largely than the hepatoprotector of the liver structure toward the complete use of lipids as energetic substance and prevents the development of the cellular metabolic mechanisms disorders in the organ, leading to structural changes.

This complies with the literature data. According to Shishkina [42], the liver microstructure of Chinese gray gees is characterized by the absence of the hepatocytes clear boundaries and fatty dystrophy. Within the endemic zone, the organic selenium preparation has a positive effect on the live weight growth of Chinese gray geese and optimizes the liver structure, helps level the processes of liver fatty degeneration due to the complete lipids use as an energy source [42]. The other authors examined selenium impact on the liver morphology, received the same data [2,4,12-14,19]. This challenging topic remains under further study [1]. Dironax effect on the growth and development of the poultry meat species has not been studied enough [12,22,43].

## Conclusion

There was the development of parenchymal (protein-fatty) and stromal-vascular dystrophies in the liver against the lack of natural antioxidants in the fodder. In our opinion, among the morphogenetic mechanisms, leading to the changes typical for the dystrophies, while in this case, the fat components transformation and membranes lipid peroxidation prevail. Endogenous metabolites accumulate with insufficient or incomplete lipid utilization. It is typical for birds. This reduces the liver compensatory capacity in relation to the effects of these factors and leads to further development of these pathological processes, up to alterative and fibrotic changes.

The performed studies allowed us to examine the positive effect of the antioxidants included in the polyvitamin selenium-containing feed supplement (Solvimin Selen) and new domestic preparation based on the diisopropylammonium dichloroacetate (Dironax) on the meat bird growth and development (Muscovy ducks, Hungarian White geese, and quails), morphofunctional liver state; therefore, we could make the following conclusions.

The use of the antioxidants adjusts the established organogenesis patterns of the birds’ liver. They optimize the organ’s structure toward the complete use of lipids as an energetic substance and prevent the development of the cellular metabolic mechanisms disorders in the organ, leading to structural changes.

The high biological and prophylactic activity of Solvimin Selen was confirmed already in the 1^st^ month of the postembryonic embryogenesis. Diisopropylammonium dichloroacetate has a hepatoprotective effect, preventing the development of hepatocytes fatty degeneration and subsequent fibrotic changes in the liver stroma, but this effect is observed later compared with Solvimin Selen.

## Authors’ Contributions

ES carried out the study and wrote the manuscript. GB, GBa, OD, and RK participated in the drafting and revision of the manuscript. All authors planned and conducted the study. All authors approved the final manuscript.
